# Predictive value of preoperative lymphocyte-to-monocyte ratio on survival outcomes in bladder cancer patients after radical cystectomy

**DOI:** 10.7150/jca.50603

**Published:** 2021-01-01

**Authors:** Hai Bi, Ye Yan, Dong Wang, Zijian Qin, Guoliang Wang, Lulin Ma, Yi Huang, Jian Lu

**Affiliations:** 1 Department of Urology, Peking University Third Hospital, 49 North Garden Road, Haidian District, Beijing 100191, PR China.; 2 Department of Urology, National Cancer Center/National Clinical Research Center for Cancer/Cancer Hospital, Chinese Academy of Medical Sciences and Peking Union Medical College, Beijing 100021, PR China.

**Keywords:** Bladder cancer, radical cystectomy, lymphocyte-to-monocyte ratio, survival outcomes, predictive accuracy

## Abstract

**Purpose:** To determine the prognostic significance of the pre-operative lymphocyte-to-monocyte (LMR) in patients with bladder cancer (BCa) who underwent radical cystectomy (RC), and to assess its prognostic benefit compared to models relying solely on clinicopathological factors.

**Materials and Methods:** A retrospective analysis of the 342 BCa patients undergoing RC at our institution from 2004 to 2017 was conducted to assess LMR prognostic significance. Overall survival (OS) and cancer-specific survival (CSS) were assessed using the Kaplan-Meier method. Cox regression models identified risk factors for survival outcomes. Two new models were developed based on basal models to predict OS and CSS at 1, 3, and 5 years after RC. The accuracy of the new models was evaluated using receiver operating characteristic curves as well as the concordance index. We also conducted decision curve analysis (DCA) to assess their net benefit.

**Results:** An association between excellent long-term patient survival outcomes and higher LMR levels was observed. The median OS and CSS for higher LMR level in patients was 98.8 months and >120 months, respectively. Cox regression multivariate analysis showed that pre-operative LMR, as a continuous variable, was an independent survival outcome predictor (*p*<0.001). The utilization of LMR in the standard model resulted in significant discriminatory improvement in OS (5.6%, *p*<0.001) and CSS (4.9%, *p*=0.001) prediction. Moreover, as shown in DCA, utilization of the new model, including LMR, resulted in a net benefit compared to base models for predicting OS and CSS at 1, 3, and 5 years.

**Conclusions:** An independent association was observed between higher pre-operative LMR in BCa patients undergoing RC and significantly better OS and CSS. In addition, a significant improvement in predictive accuracy was observed with LMR inclusion in multiparametric prediction tools. Therefore, LMR may be utilized in pre-operative patient risk stratification to assist in patient counseling and clinical decision making.

## Introduction

Bladder cancer (BCa) is the second most common genitourinary tract malignancy worldwide, with approximately 80,470 new cases and 17,670 cancer-related deaths reported in 2019 1. Radical cystectomy (RC) remains the standard therapeutic approach for non-metastatic muscle-invasive BCa as well as high-risk non-muscle-invasive BCa 2, whereas the 5-year mortality rates of patients with BCa after RC are as high as 38-46% 3-4. Furthermore, despite multimodal treatment approaches, survival outcomes after RC have not changed in the past several decades 5. Thus, improvement in the accuracy of pre-operative risk stratification is warranted.

Risk assessment and estimating survival after RC are essential for patient counseling and the design of treatment regimens. The American Joint Committee on Cancer (AJCC) tumor-node-metastasis (TNM) staging system is the most commonly employed prediction tool 6. Recently, several models for BCa patient assessment have been created using clinicopathological features and are reportedly more accurate at prediction compared to the TNM staging system 7. A classical study identified variables such as age, pathologic TNM staging system, pathologic tumor grade, presence of lymphovascular invasion (LVI) at RC, as well as administration of adjuvant therapy (chemotherapy or radiotherapy) to be predictive of survival outcomes after RC, which constitute Bladder Cancer Research Consortium (BCRC) models 8. However, the majority of predictive variables are post-operative pathologic features, and the accuracy of BCa clinical staging remains relatively low 9. Thus, novel prognostic markers are required to facilitate appropriate pre-operative patient counseling.

Systemic inflammatory responses are important prognostic elements for the development and progression of cancer 10. They are particularly relevant in BCa, which is a highly immunogenic malignancy 11. Several studies have identified the lymphocyte-to-monocyte ratio (LMR) as an independent prognostic factor for post-RC survival outcomes 12-17. However, the contribution of LMR in improving the prognostic accuracy of established BCa outcome predictors remains unclear 17, which can be addressed by measurement of incremental predictive accuracy 18 and decision curve analysis (DCA) 19.

Thus, this study was designed to assess the prognostic significance of pre-operative LMR in BCa patients who underwent RC, and to evaluate its prognostic benefit compared to models that rely on clinicopathological factors alone.

## Materials and Methods

### Patient population

This study utilized the BCa database of the Department of Urology of Peking University Third Hospital (PUTH; Beijing, China), with approval from the Institutional Review Board for the Protection of Human Subjects. 377 consecutive BCa patients who underwent RC between 2004 and 2017 at PUTH were included in the study. For each patient, comprehensive clinical and pathologic information was reviewed and collected. Of the 377 patients, 35 were excluded for the following reasons: 12 due to pathologic cell type other than urothelial carcinoma, 4 due to distant metastasis disease at the time of RC, 8 due to post-operative 30-day death, and 11 due to conditions before RC that could affect blood cell lines (systemic inflammatory disease: n = 7, malignant lymphoma: n = 2, neoadjuvant chemotherapy: n = 2). This resulted in 342 BCa patients eligible for further analysis. All patients received laparoscopic RC. Extension of pelvic lymph node dissection (PLND) and urinary diversion type were based on the surgeon's discretion. Regarding urinary diversion, 170 cases underwent ureterocutaneostomy, 122 cases underwent ileal conduit, and 50 cases received orthotopic neobladder. A standard PLND was conducted in the majority of patients, except 12 cases in which extended PLND was performed.

### Study variables

Study variables were extracted from the database and included age, gender, pathologic TNM staging system, pathologic tumor grade, presence of LVI, administration of adjuvant therapy (chemotherapy or radiotherapy), and LMR, according to the BCRC models 8. RC pathologies were reviewed by staff pathologists with expertise in genitourinary pathology for tumor stage, tumor grade, and presence of LVI. BCa was staged based on the criteria listed in the AJCC staging manual, 7^th^ edition. Tumor grade was determined using the 2004 World Health Organization grading system. LVI was described as the presence of tumor cells that were nested within an endothelium-lined space and in the absence of underlying muscular walls 20. Routine complete blood counts were collected within 30 days of RC as part of the routine pre-operative clinical assessment. The LMR was calculated using the formula: the lymphocyte count (10^9^/L) divided by the monocyte count (10^9^/L). Clinical notes were reviewed to rule out any signs or symptoms of infection around the time of blood test. Patient survival outcome correlations were evaluated using LMR as a continuous variable, with an LMR cut-off point described as the median value 3.27.

### Follow-up

Generally, the patient underwent clinical and radiological follow-up postoperatively following routine institutional protocol. The follow-up consisted of quarterly sessions for the first 2 years, semiannually for the next 2 years, and then annual follow-up thereafter. Study outcomes included cancer-specific survival (CSS) and overall survival (OS) relative to the time of treatment initiation.

### Statistical analysis

Descriptive statistics were performed using medians and interquartile ranges (IQRs) for continuously coded variables. For categorical variables, frequencies and proportions were utilized. The chi-square and independent samples Mann-Whitney U tests were employed to assess the statistical significance of differences among proportions and means. CSS and OS were calculated using the Kaplan-Meier approach. Survival between patients with an LMR < 3.27 and ≥ 3.27 were compared using the log-rank test. The Cox proportional-hazards model was employed to evaluate the correlation between LMR and survival outcomes and by controlling for clinicopathological variables. In particular, the TNM models consisted of pathologic T and N stages, whereas the BCRC models comprised age, pathologic T and N stages, tumor grade, LVI presence, and adjuvant therapy. Based on the BCRC models, we generated two new prognostic models that included LMR for CSS and OS. To test the discriminatory ability of the models, we employed Harrell's concordance (C) index 21. Approximately 1,000 bootstrap resamples were employed for internal validation, and calibration plots were constructed to assess the new models' performance 22. The predictive accuracy of three models at 1, 3, and 5 years was calculated using time-dependent receiver operating characteristic curve (ROC)-derived area under the curve (AUC) estimates 23. The predictive abilities of the three models were compared using Kang's method 24. Finally, to determine the net increase in the proportion of cases that were identified by the new models, the DCA was calculated at 1, 3, and 5 years 19. All statistical analyses were performed using R (the R Foundation for Statistical Computing, Vienna, Austria, ver. 3.6.1). Differences were deemed statistically significant at *p*<0.05.

## Results

### Clinicopathological variables of patients

Table 1 shows the characteristics of the 342 BCa patients included in this study. The median patient age was 68 years (IQR 59-75 years), and the median pre-operative LMR level was 3.27 (IQR: 2.42-4.25). Clinicopathological factors for patients with pre-operative LMR ≥ 3.27 and < 3.27 are also provided in Table 1. Patients with an LMR < 3.27 were significantly older (*p*=0.023) and more likely at RC to have pathologic extravesical (pT3/4) tumor (45.6% *vs.* 25.1%; *p*<0.001) as well as lymph node tumor involvement (21.6% *vs.* 9.9%; *p*=0.005).

### OS and CSS in the cohort

**Figure 1:**
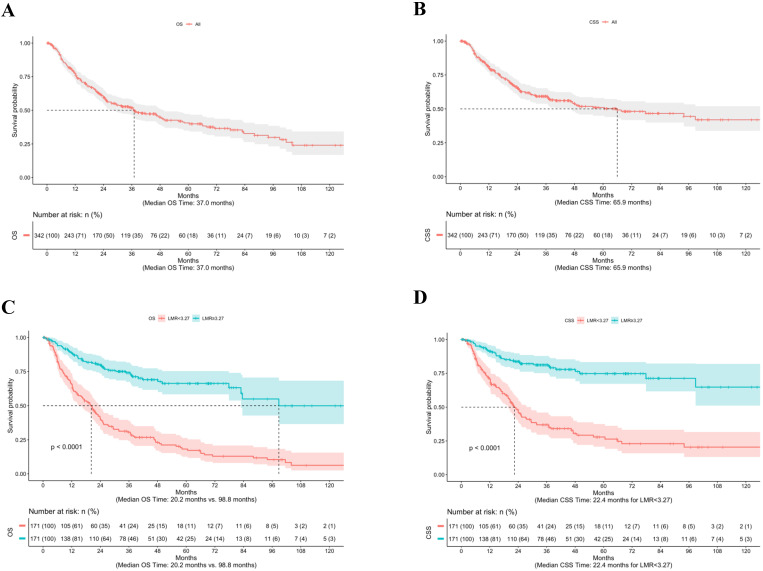
Kaplan-Meier estimates of OS (**A**) and CSS (**B**) for 342 BCa patients treated with RC, as well as OS (**C**) and CSS (**D**) according to LMR ≥3.27 and <3.27. **Abbreviation:** BCa: bladder cancer; CSS: cancer-specific survival; LMR: lymphocyte-to-monocyte ratio; OS: overall survival; RC: radical cystectomy.

The median follow-up from time of RC was 23.8 months (mean 32.9, range 0.3-167.4). Overall, 183 patients (53.5%) died of any cause, of which 139 deaths (76.0%) were due to BCa. The median OS time was 37.0 months (Fig. 1A), whereas the median CSS time was 65.9 months (Fig. 1B). Figure 1 shows the stratified Kaplan-Meier estimators as well as the significantly different OS (Fig. 1C) and CSS (Fig. 1D) rates based on an LMR ≥ 3.27 and < 3.27 (both log-rank *p*<0.001).

### Independent prognostic factors for OS

We assessed the association between pre-operative LMR and OS outcomes using multivariate Cox regression analysis by controlling for other clinicopathological characteristics (Table 2). We observed that when LMR was evaluated as a continuous variable, it was independently correlated with a significantly better OS (hazards ratio [HR]: 0.549; *p*<0.001), i.e., for every one-unit increase in LMR, there was a 45.1% reduction in the risk of all-cause death. Furthermore, multivariate analysis indicated that except for LMR, a significant association of patient age, pathologic tumor stage, and lymph node status with OS was observed (Table 2).

### Prognostic new model for OS

**Figure 2:**
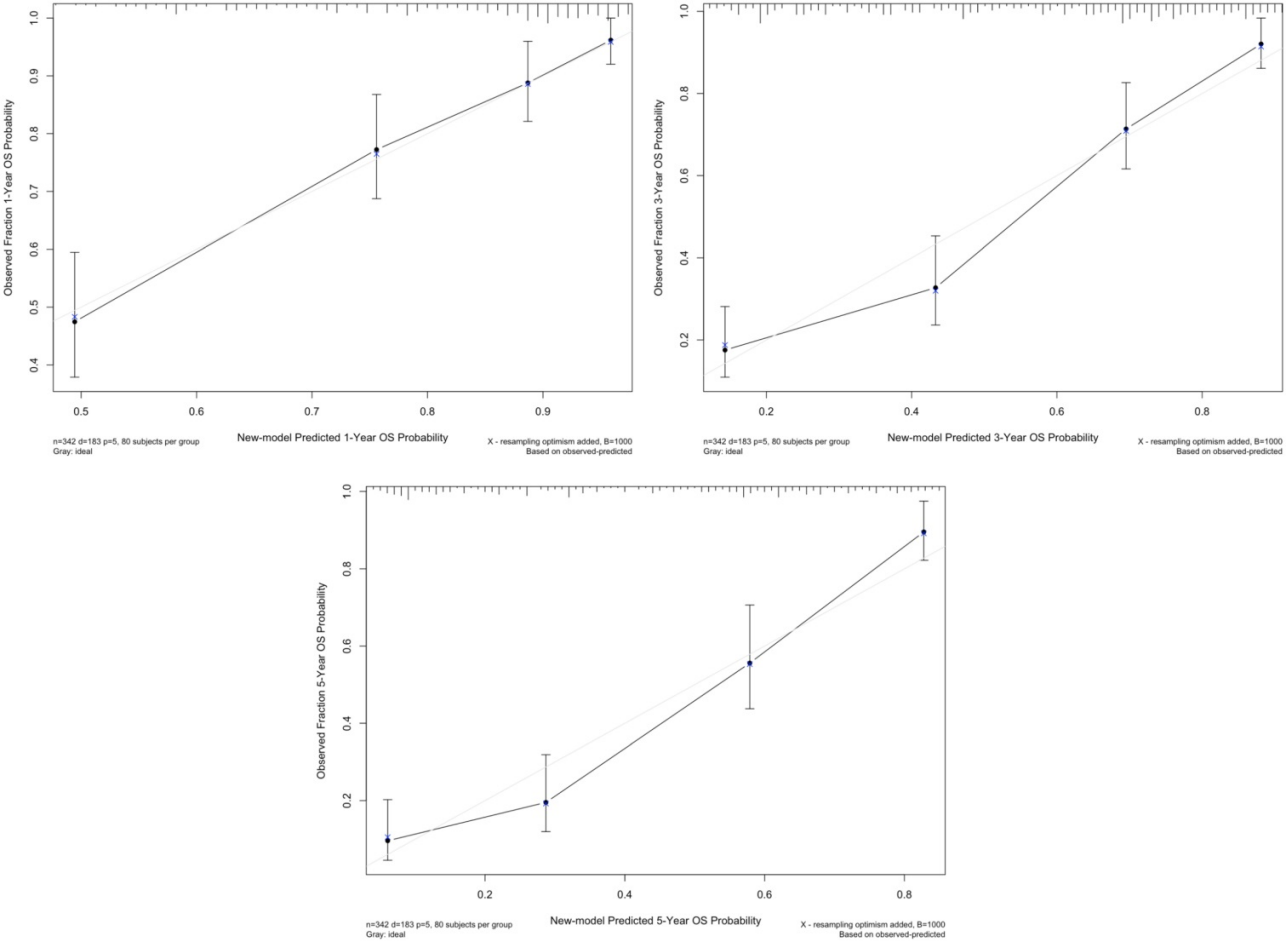
The calibration curve of new model for predicting patients' OS at 1 year, 3 years, and 5 years. **Abbreviation:** OS: overall survival.

According to the multivariate analysis, we generated a new prognostic model that included LMR for OS, which showed a C-index of 0.771 (95% confidence interval [CI]: 0.736-0.806), whereas the C-indices of the TNM model and BCRC model were 0.674 (95% CI: 0.636-0.712) and 0.715 (95% CI: 0.676-0.754), respectively (both *p*<0.001). The calibration plot for OS probability revealed optimal agreement between new model's prediction and actual observation at all indicated time points (Fig. 2).

### Comparison of OS predictive accuracy in new model and conventional predictive models

**Figure 3:**
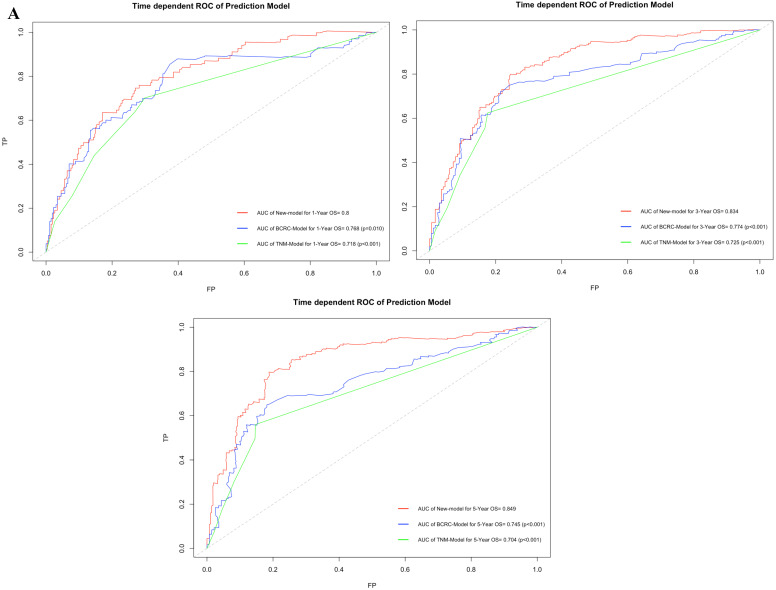

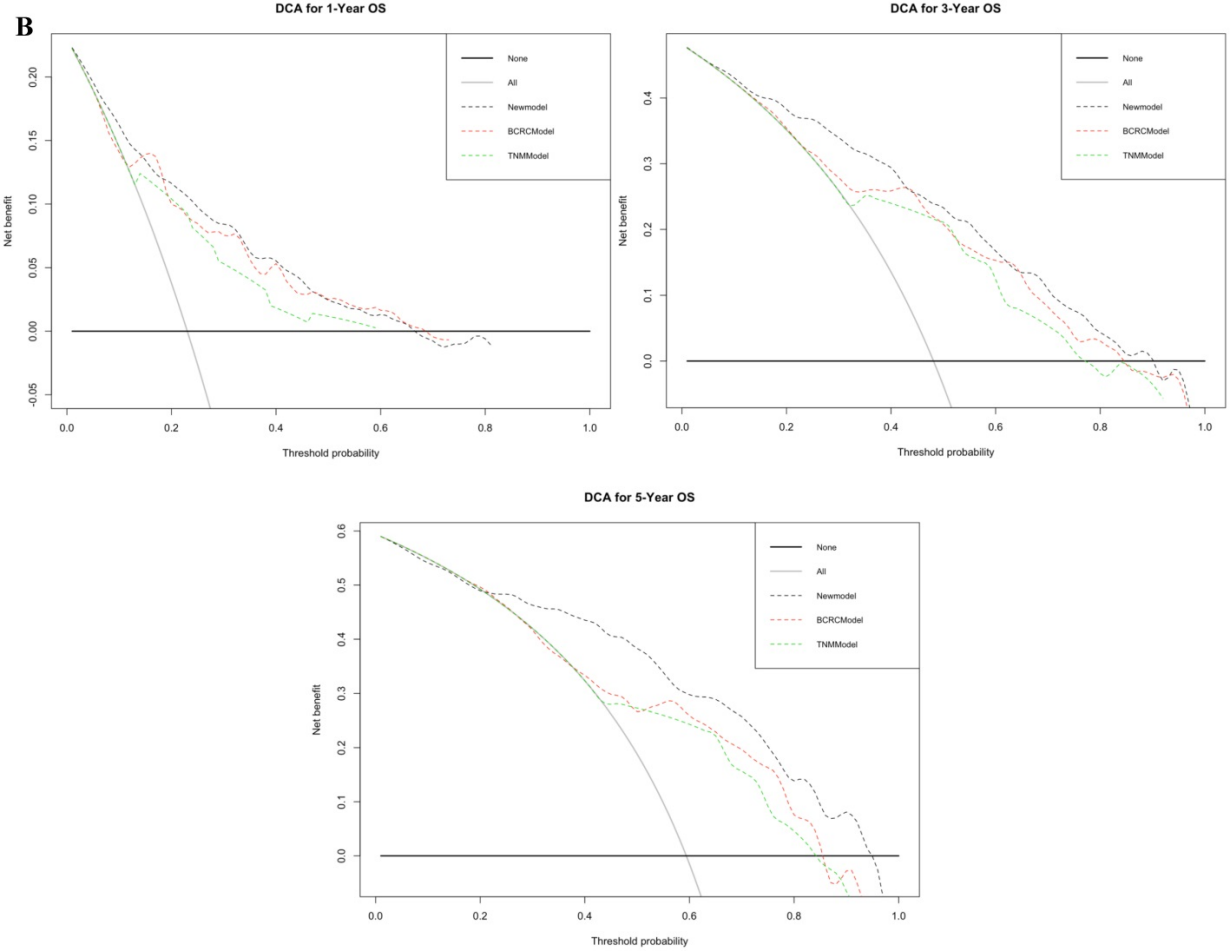
** A.** In time-dependent ROC, the AUC of new model for OS were all significantly superior to TNM-Model and BCRC-Model at one, three, and five years after RC in BCa patients. **B.** In DCA, the new model appeared to confer an advantage in predicting OS at one, three, and five years after RC. **Abbreviation:** AUC: area under curve; BCa: bladder cancer; BCRC: Bladder Cancer Research Consortium; DCA: decision curve analysis; OS: overall survival; RC: radical cystectomy; ROC: receiver operating characteristic curve.

Figure 3A shows that using time-dependent ROC, the AUCs of the new model were 0.800, 0.834 and 0.849 at 1, 3, and 5 years after RC, whereas those of the BCRC model were 0.768 (*p*=0.010), 0.774 (*p*<0.001), and 0.745 (*p*<0.001), and of the TNM model were 0.718 (*p*<0.001), 0.725 (*p*<0.001), and 0.704 (*p*<0.001) at 1, 3, and 5 years, respectively. These differences in AUC were determined to be statistically significant. Similarly, compared to the TNM model and BCRC model, DCA revealed that the new model had a net clinical benefit across a specific range of clinically relevant OS threshold probabilities at the indicated time points (Fig. 3B).

### Independent prognostic factors for CSS

We evaluated the correlation between pre-operative LMR and CSS outcomes by multivariate analysis, controlling for other clinicopathological characteristics (Table 3). We observed that LMR, when evaluated as a continuous variable, remained independently correlated with a significantly better CSS (HR: 0.569, *p*<0.001), i.e., for every one-unit LMR increase, there was a 43.1% reduction in the risk of cancer-specific death. Furthermore, multivariate analysis showed that the pathologic tumor stage and lymph node status were similarly significantly associated (Table 3).

### Prognostic new model for CSS

**Figure 4:**
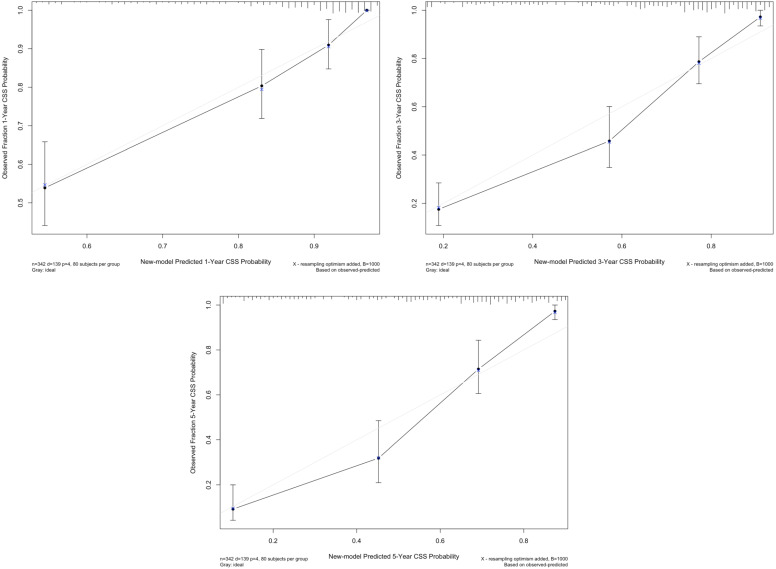
The calibration curve of new model for predicting patients' CSS at 1 year, 3 years, and 5 years. **Abbreviation:** CSS: cancer-specific survival.

We used multivariate regression coefficients to generate a new prognostic model for CSS, which showed a C-index of 0.804 (95% CI: 0.770-0.838), whereas the TNM model was 0.743 (95% CI: 0.704-0.782; *p*<0.001) and the BCRC model was 0.755 (95% CI: 0.715-0.795; *p*=0.001). The calibration plot for CSS probability revealed an optimal agreement between new model prediction and actual observation at 1, 3, and 5 years after RC (Fig. 4).

### Comparison of CSS predictive accuracy in new model and conventional predictive models

**Figure 5:**
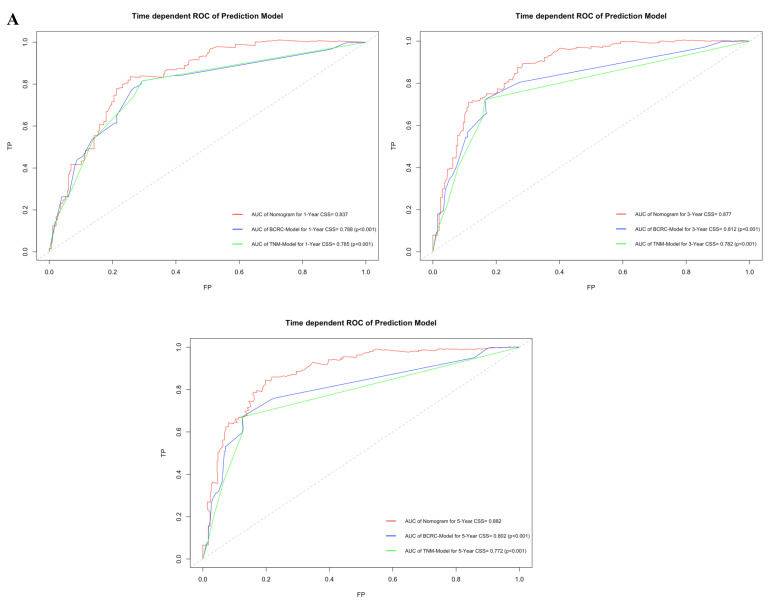

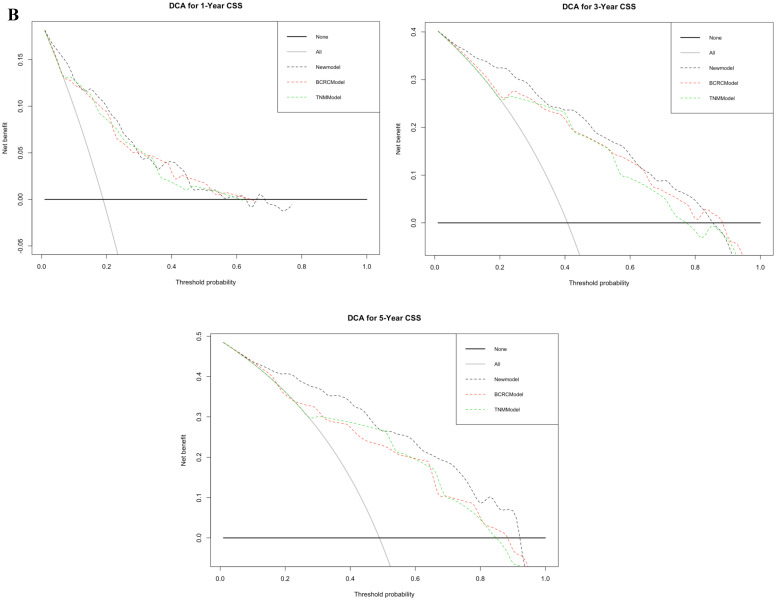
** A.** In time-dependent ROC, the AUC of new model for CSS were all significantly superior to TNM-Model and BCRC-Model at one, three, and five years after RC in BCa patients. **B.** In DCA, the new model appeared to confer an advantage in predicting CSS at one, three, and five years after RC. **Abbreviation:** AUC: area under curve; BCa: bladder cancer; BCRC: Bladder Cancer Research Consortium; DCA: decision curve analysis; CSS: cancer-specific survival; RC: radical cystectomy; ROC: receiver operating characteristic curve.

By comparing time-dependent ROCs, the AUCs of the new model were 0.837, 0.877 and 0.882 at 1, 3, and 5 years after RC, whereas those of the BCRC model were 0.785 (*p*<0.001), 0.782 (*p*<0.001), and 0.772 (*p*<0.001), and of the TNM model were 0.788 (*p*<0.001), 0.812 (*p*<0.001), and 0.802 (*p*<0.001) at 1, 3, and 5 years, respectively (Fig. 5A). These differences in AUC were all statistically significant. Similarly, compared to the TNM model and BCRC model, DCA revealed that the new model had a net clinical benefit across a specified range of clinically relevant CSS threshold probabilities at the indicated time points (Fig. 5B).

## Discussion

This study retrospectively evaluated relevant data from 342 BCa patients who underwent RC to establish the relationship between pre-operative LMR and survival outcomes. A direct correlation between lower pre-operative LMR and advanced pathologic tumor stage and lymph node status during RC was observed, as well as lower OS and CSS probability. Multivariate analysis revealed that pre-operative LMR was an independent OS and CSS prognostic factor after controlling for clinicopathological characteristics. Patients with greater LMR had better outcomes.

Lymphocyte and monocyte counts, which are components of the pre-operative blood test, are inexpensive and readily available in the clinic, and thus may be an optimal diagnostic and prognostic biomarker. Furthermore, LMR collected before all treatments (e.g., cystoscopy), without any influence, is preferred. LMR, at this point in time, merely represents the complex interactions among patient frailty, competing comorbidities, and locally advanced disease 25. Although several researchers have indicated that high pre-treatment LMR predicts good cancer patient outcomes 26, 27, there is still no consensus on the ideal cut-off value. Thus, we evaluated LMR as a continuous variable in Cox regression analysis.

The correlation between increased pre-operative LMR and patient survival outcomes is relatively complex and needs further investigation. BCa is frequently associated with chronic or recurrent urinary tract inflammation and systemic inflammation 11, 28. In turn, via oncogenic changes, the tumor microenvironment activates the adaptive immune response that in turn induces cancer-promoting inflammation that promotes proliferation, progression, and metastasis 29. Monocytes play a major role in proinflammatory cytokine production, which includes monocyte chemoattractant protein-1 that contributes to cancer initiation and promotion 30. Additionally, high tumor-associated macrophage counts are correlated with poor survival and poor response to treatment 31. However, lymphocytes are essential in antitumor reactions through the induction of tumor cell apoptosis 32 and by mediating antibody-dependent cell-mediated cytotoxicity 33. In studies of BCa, the number and function of lymphocytes have both been found to be lower in invasive disease than in controls and superficial carcinoma 31, 34. Thus, a low LMR indicates a weaker, lymphocyte-mediated, antitumor immune response as well as more extensive inflammation and monocyte-mediated immune dysfunction 35.

To date, five studies have addressed the role of pre-treatment LMR in the survival of BCa patients who underwent RC. Temraz *et al.* 12 found that based on the cut-off value of 2.81, patients with lower level LMR had shorter OS (2.7 vs. 6.0 years, *p*=0.020) in a 68-BCa patient cohort. There was no multivariable analysis in this study. Zhang* et al.* 13 assessed LMR prognostic value in 124 BCa patients who were treated with RC. After adjusting for confounding factors, patients with an LMR ≥ 4 indicated >30% decreased mortality than the low LMR group (*p*=0.003). Rajwa *et al.* 16 evaluated LMR as a continuous factor in multivariate analysis, and found that lower LMR was associated with a shorter CSS (HR: 0.752; *p*=0.0006) and OS (HR: 0.785; *p*=0.001). In these two studies, no assessment of specific relevant confounders (e.g., LVI, adjuvant chemotherapy/radiotherapy, and/or concomitant systemic inflammatory disease) was performed. Furthermore, Yoshida *et al.* 14, 15 reported that pre-operative LMR has higher predictive accuracy for OS compared to other inflammatory markers (*p*=0.033), and interestingly, perioperative LMR changes were significantly associated with OS (HR: 5.70; *p*<0.001) and CSS (HR: 4.53; *p*<0.001). However, there were no studies that assessed whether LMR enhances the prognostic accuracy of established BCa outcome predictors. The largest multicenter study to date, which was conducted by D'Andrea *et al.* 17, confirmed that LMR can independently predict CSS and OS, and the discriminatory power of the nomogram increases with LMR, although not significantly. Therefore, we utilized the C-index comparison, time-dependent ROC, and DCA to evaluate the prognostic benefit conferred by LMR.

Several findings in this study are noteworthy. First, we revealed excellent long-term survival outcomes among patients with higher LMR levels. The median survival time for higher LMR level patients was 98.8 months in OS and > 120 months in CSS. In Cox multivariate analysis, pre-operative LMR, as a continuous variable, is an independent survival outcome predictor, which avoids the need to determine the ideal cut-off value 35. Similar to D'Andrea *et al.* 17, we added the LMR to the standard models to validate the improvement of prognostic accuracy. The inclusion of LMR into our new model induced a significant improvement in OS and CSS prediction at all determined time points (*p*<0.01). In addition, as shown in the DCA, the use of LMR in new models confers a net benefit compared to the base models in predicting OS and CSS. Hence, LMR may be potentially added to the basal models for predicting OS and CSS in BCa patients post-RC and to guide in the design of adjuvant treatment schemes.

This study had many limitations that were mainly related to its retrospective and single-institutional nature, possibly resulting in selection bias and discrepancies from other geographical regions as well as institutions. Therefore, in the future, a large prospective cohort and multicenter study are needed to investigate the role of LMR in survival outcomes of BCa patients after RC. In this cohort, the calibration plot for the survival outcomes probability revealed an optimal agreement at various time points; however, we did not perform any external validation. The predictive accuracy of our new models in other cohorts should thus be assessed. In addition, the role of LMR in survival outcomes for BCa can be applied to patients with non-urothelial carcinoma of BCa should also be investigated.

## Conclusions

Lower pre-operative LMR was correlated with advanced tumor stage in BCa patients who underwent RC. After controlling for clinicpathological factors, increased LMR remained associated with a significantly better OS and CSS. Moreover, a significant increase in predictive accuracy was observed after LMR inclusion into multiparametric prediction tools. In addition, LMR is inexpensive and readily available in routine pre-operative testing. Therefore, it may be useful in pre-operative patient risk stratification to help patient counseling and clinical decision making.

## Figures and Tables

**Table 1:** Perioperative characteristics of 342 BCa patients treated with RC between 2004 and 2017

Variables	Overall (n=342)	LMR<3.27 (n=171)	LMR≥3.27 (n=171)	*p*
Age (years), Median (IQR)	68 (59-75)	70 (61-76)	66 (58-73)	**0.023**
**Gender, n (%)**				0.057
Male	286 (83.6%)	150 (87.7%)	136 (79.5%)	
Female	56 (16.4%)	21 (12.3%)	35 (20.5%)	
LMR, Median (IQR)	3.27 (2.42-4.25)	NA	NA	NA
**pT, n (%)**				**<0.001**
≤T2	221 (64.6%)	93 (54.4%)	128 (74.9%)	
T3	71 (20.8%)	45 (26.3%)	26 (15.2%)	
T4	50 (14.6%)	33 (19.3%)	17 (9.9%)	
**pN, n (%)**				**0.005**
Negative	288 (84.2%)	134 (78.4%)	154 (90.1%)	
Positive	54 (15.8%)	37 (21.6%)	17 (9.9%)	
**Pathological Grade, n (%)**				0.226
LG	18 (5.3%)	6 (3.5%)	12 (7.0%)	
HG	324 (94.7%)	165 (96.5%)	159 (93.0%)	
**LVI, n (%)**				0.089
Absent	222 (64.9%)	103 (60.2%)	119 (69.6%)	
Present	120 (35.1%)	68 (39.8%)	52 (30.4%)	
Adjuvant Therapy*, n (%)				1.000
No	295 (86.3%)	147 (86.0%)	148 (86.5%)	
Yes	47 (13.7%)	24 (14.0%)	23 (13.5%)	

**Abbreviation:** BCa: bladder cancer; HG: high grade; IQR: interquartile ranges; LG: low grade; LMR: lymphocyte-to-monocyte ratio; LVI: lymphovascular invasion; NA: not applicable; pN: pathologic node stage; pT: pathologic tumor stage; RC: radical cystectomy. * Adjuvant radiotherapy and/or adjuvant chemotherapy.

**Table 2:** Univariate and multivariate Cox regression analysis for prediction of OS after RC for BCa

Variables	Univariate Analysis			Multivariate Analysis	
	HR (95% CI)	*p*	C-index	HR (95% CI)	*p*
Age (years)	1.033 (1.017-1.049)	**<0.001**	0.608	1.031 (1.015-1.046)	**<0.001**
**Gender**			0.497		
Male	Ref				
Female	0.899 (0.597-1.353)	0.608			
LMR	0.506 (0.438-0.584)	**<0.001**	0.707	0.549 (0.475-0.636)	**<0.001**
**pT**			0.654		
≤T2	Ref			Ref	
T3	2.786 (1.966-3.947)	**<0.001**		1.746 (1.207-2.525)	**0.003**
T4	4.178 (2.845-6.134)	**<0.001**		2.567 (1.704-3.866)	**<0.001**
**pN**			0.582		
Negative	Ref			Ref	
Positive	3.094 (2.187-4.375)	**<0.001**		1.801 (1.245-2.604)	**0.002**
**Pathological Grade**			0.522		
LG	Ref			Ref	
HG	3.564 (1.320-9.623)	**0.012**		1.315 (0.477-3.621)	0.596
**LVI**			0.592		
Absent	Ref			Ref	
Present	2.236 (1.656-3.018)	**<0.001**		1.177 (0.817-1.696)	0.382
Adjuvant Therapy*			0.589		
No	Ref				
Yes	1.055 (0.704-1.581)	0.795			

**Abbreviation:** BCa: bladder cancer; CI: confidence interval; HG: high grade; HR: Hazards ratio; LG: low grade; LMR: lymphocyte-to-monocyte ratio; LVI: lymphovascular invasion; OS: overall survival; pN: pathologic node stage; pT: pathologic tumor stage; RC: radical cystectomy; Ref: reference. * Adjuvant radiotherapy and/or adjuvant chemotherapy.

**Table 3:** Univariate and multivariate Cox regression analysis for prediction of CSS after RC for BCa

Variables	Univariate Analysis			Multivariate Analysis	
	HR (95% CI)	*p*	C-index	HR (95% CI)	*p*
**Gender**		0.431	0.500		
Male	Ref				
Female	0.823 (0.507-1.336)				
LMR	0.496 (0.420-0.586)	**<0.001**	0.714	0.569 (0.481-0.674)	<0.001
**pT**			0.713		
≤T2	Ref			Ref	
T3	4.258 (2.849-6.362)	**<0.001**		2.638 (1.716-4.055)	<0.001
T4	6.920 (4.531-10.570)	**<0.001**		4.106 (2.608-6.465)	<0.001
**pN**			0.619		
Negative	Ref			Ref	
Positive	4.335 (3.007-6.251)	**<0.001**		1.957 (1.328-2.885)	0.001
**Pathological Grade**			0.531		
LG	Ref			Ref	
HG	10.340 (1.444-73.980)	**0.020**		3.576 (0.492-25.997)	0.208
**LVI**			0.631		
Absent	Ref			Ref	
Present	3.209 (2.283-4.510)	**<0.001**		1.378 (0.918-2.069)	0.122
**Adjuvant Therapy**			0.505		
No	Ref				
Yes	1.424 (0.939-2.158)	0.096			

**Abbreviation:** BCa: bladder cancer; CI: confidence interval; HG: high grade; HR: Hazards ratio; LG: low grade; LMR: lymphocyte-to-monocyte ratio; LVI: lymphovascular invasion; CSS: cancer-specific survival; pN: pathologic node stage; pT: pathologic tumor stage; RC: radical cystectomy; Ref: reference. * Adjuvant radiotherapy and/or adjuvant chemotherapy.
